# Comparative Determination of Glomerular Filtration Rate Estimation Formulae in Type 2 Diabetic Patients: An Observational Study

**DOI:** 10.1155/2024/9532236

**Published:** 2024-06-10

**Authors:** Emmanuel Kwaku Ofori, Irene Nketiah-Dwomo, Emmanuel Aryee Tagoe, Seth Kwabena Amponsah, Ismaila Adams, Eric Nana Yaw Nyarko, Seth Dortey Amanquah

**Affiliations:** ^1^ Department of Chemical Pathology U.G.M.S. University of Ghana, Accra, Ghana; ^2^ Department of Medical Laboratory Narh-Bita College, Tema, Ghana; ^3^ Department of Medical Laboratory S.B.A.H.S. University of Ghana, Accra, Ghana; ^4^ Department of Medical Pharmacology U.G.M.S. University of Ghana, Accra, Ghana

## Abstract

Assessing glomerular filtration rate (GFR) involves collecting timed urine samples for 24 hours, requiring significant time and resources in the clinical setting. Using predictive GFR formulae to assess renal function may be a better alternative. Our goal was to determine which predictive GFR formula had the highest level of concordance with the GFR that has been measured in a resource-poor setting. This is an observational study. We selected fifty (50) individuals diagnosed with type 2 diabetes (T2DM) in Kumasi, Ghana. The sociodemographic and clinical characteristics were obtained using a structured questionnaire. Urine was obtained from each subject over 24 hours. The levels of glucose (FBG) and creatinine in patients' blood, as well as the levels of creatinine in their urine, were measured after the patients had fasted overnight. Participants had a mean age of 57.4 ± 10.7 (years), BMI of 27.8 ± 4.1 (kg/m^2^), FBG of 9.0 ± 3.1 (mmol/L), and creatinine concentrations of 95.6 ± 29.1 (*μ*mol/L). A Krouwer plot was used to compare the measured GFR with three formulae: Chronic Kidney Disease Epidemiology (CKD-EPI), Modification of Diet in Renal Disease (MDRD), and Cockroft-Gault (CG) for GFR prediction. Among the 3 estimates, CG showed nonsignificance (*p* > 0.05) with the measured GFR. The primary finding was that the GFR calculated using the CG formula was not different from the GFR measured, suggesting that CG is the most appropriate alternative GFR estimate among a cross-section of T2DM patients in Ghana.

## 1. Introduction

Diabetes mellitus (DM), which is commonly referred to as diabetes, is a heterogeneous group of disorders characterized by abnormalities in carbohydrate, protein, and lipid metabolism. DM presents with abnormality in insulin production, action, or both, although other factors can be involved. DM is directly or indirectly responsible for the deaths of over 1.6 million people worldwide [1, 2]. More than 70% of people who are diagnosed with DM reside in resource-poor countries [3]. The cost involved in managing DM cannot be overemphasized [4, 5]. Genetic predisposition and an unhealthy modern lifestyle are known to be risk factors for the development of type 2 diabetes (T2DM) [6, 7]. In addition to hyperglycemia, other metabolic features of T2DM include insulin resistance (IR), obesity, hypertension, and dyslipidemia [8, 9].

In high-income countries, the most common reason for kidney failure is diabetic nephropathy (DN), often known as diabetic kidney disease [10, 11]. Increased albumin excretion has long been considered the initial clinical sign of DN among individuals with either type 1 or 2 DM [12, 13]. Survival rates of DN are low among DM patients due to cardiovascular and/or renal system-associated complications of the disease [14]. For this reason, poor renal clearance and a rise in plasma creatinine levels should be of concern to DM patients.

Glomerular filtration rate (GFR) is an important parameter in the monitoring and management of DM. Even though this parameter is considered to be one of the “standards” for measuring renal function [15], estimating creatinine clearance by collecting urine for 24 hours is often a challenge for the patient and laboratory personnel [16]. Additionally, collecting 24-hour urine is time-consuming and could delay relevant clinical decision-making [17]. Therefore, evaluating renal function in patients with T2DM using predictive GFR formulas might be a better alternative. These eGFR formulas have been validated in multiple populations [18, 19] and disease conditions [20, 21]. However, there are certain peculiarities, with each formula having advantages and disadvantages.

These equations appear population-driven, making it very important to select and validate the eGFR best suited for the population of interest and the disease under consideration. The usefulness of GFR estimation within the DM management framework has received very little attention in Ghana and other resource-poor settings. Clinical settings in Ghana utilize both the measured and estimated GFRs. The estimated GFR is not always used in the same way. Clinicians and other medical professionals decide on the estimated GFR they see fit. To our knowledge, there is no set protocol for this. While acknowledging the need for more validation, this study offers preliminary information on which estimated GFR formula is analogous to measured GFR and best applies to our male and female diabetes communities in Ghana. This knowledge is pertinent, particularly in our district and localized towns with limited resources.

We hypothesize that the measured GFR of patients with DM is comparable with the predictive GFR formulae which include the Chronic Kidney Disease Epidemiology Collaboration (CKD-EPI), Modification of Diet in Renal Disease (MDRD), and Cockroft-Gault (CG).

## 2. Materials and Methods

### 2.1. Study Design, Participants, Site, Minimum Sample Size, and Ethical Consideration

An observational study design was used. The sampling strategy employed was both convenient and purposive. The study was carried out from April to August 2019. Fifty (18 men and 32 women) community-dwelling individuals diagnosed with T2DM within the last 5 years without known DM complications and attending the Diabetes Management Clinic at Suntreso Government Hospital, Kumasi, Ghana, were recruited after obtaining informed consent. The ages of participants ranged from 30 to 70 years old. Women who were pregnant when they were diagnosed with diabetes, persons who were infected with human immunodeficiency virus (HIV) or hepatitis B, patients who had a chronic renal disorder or who required dialysis, wheelchair-bound individuals, heavy smokers, and those who had experienced a stroke or amputation were excluded from this study. The above are known to affect creatinine levels. Participants were assisted in completing a standardized questionnaire that inquired about their sociodemographic and clinical characteristics.

To obtain the necessary sample size power (80%) in this study, there needed to be a minimum of 35 study participants. This was based on the premise that the predicted correlation coefficient was 0.50 when the significance level was set at 5%. Before initiating the study, approval had to be received from the Ethical Committee of the Allied Health Sciences, Narh-Bita College, and the management of the South Suntreso Government Hospital.

### 2.2. Clinical Assessment

After the participants gave content, they were asked to remove their shoes or boots, and their heights in centimeters were measured with a stadiometer that was fixed on the wall (Secca, Germany). To determine body weight, a typical digital bathroom scale was used (Tanita Corporation, Tokyo, Japan). After dividing the body weight by a square of the height, the body mass index (BMI) was calculated for each individual.

### 2.3. Biochemical Analysis

After the subjects had fasted for 10-14 hours following the Helsinki protocol declaration [22], 4 mL of the participants' venous blood was collected between the hours of 7:00 and 9:00 am. One milliliter (1 mL) of whole blood was put in a tube containing sodium fluoride and centrifuged, with the resulting plasma separated for fasting plasma glucose (FPG) measurement. After transferring the remaining 3 mL of the blood sample into a serum separator tube, it was then processed by centrifugation, and the resulting sera were frozen at a temperature of -20°C until the analysis. An autoanalyzer (Roche-Hitachi Modular Analytics, Tokyo, Japan) was used to perform the biochemical analysis.

In addition, urine samples were collected from participants for 24 hours and placed in disposable, clean, dry, and sterile plastic universal containers that prevented leaks. Before commencing the timed collection, the participants were instructed to empty their bladders. This was done to collect the 24-hour urine sample as precisely as feasible. After that, all of the urine was put together and placed in a container, where it stayed undisturbed until the final collection, which took place at the very end of the 24 hours. The urine specimen was refrigerated at 4°C during the collection period. The specimen container was returned to the laboratory the same morning after collection.

### 2.4. Measured GFR

Measured GFR was calculated based on the formula below:

(1) $$\begin{array}{c} \text{Measured GFR}=\frac{U\times V}{P}\text{mL}/\min, \end{array}$$

where *U* is the urine creatinine concentration, *V* is the urine flow rate (mL), and *P* is the plasma creatinine concentration (*μ*mol/L).

Measured GFR was corrected to a standard body surface area (BSA) of 1.73 m^2^ using a formula that incorporates weight and height to allow easier comparison between individuals [23].

(2) $$\begin{array}{c} \text{Corrected measured GFR}=\frac{\text{uncorrected measured creatinine clearance}\times 1.73}{{\text{weight}}^{0.425}\times {\text{height}}^{0.725}\times 0.007184}, \end{array}$$

(3) $$\begin{array}{c} \text{Corrected measured GFR}=\frac{\left(U\times V/P\right)\times 1.73}{{\text{weight}}^{0.425}\times {\text{height}}^{0.725}\times 0.007184}. \end{array}$$

### 2.5. Other Formulas of GFR Estimation

These 3 formulae were also used to estimate the GFR.

### 2.6. MDRD

175 × (plasma creatinine)^−1.154^ × age^−0.203^ (×0.742 if female; ×1.21 if black) [24].

### 2.7. CKD-EPI

(Plasma creatinine/0.7)^−0.329^ × 0.993^age^ (×166), for women with a plasma creatinine ≤ 62 *μ*mol/L.

(Plasma creatinine/0.7)^−1.209^ × 0.993^age^ (×166), for women with a plasma creatinine > 62 *μ*mol/L.

(Plasma creatinine/0.9)^−0.411^ × 0.993^age^ (×163), for men with a plasma creatinine ≤ 80 *μ*mol/L.

(Plasma creatinine/0.9)^−1.209^ × 0.993^age^ (×166), for men with a plasma creatinine > 80 *μ*mol/L [25]

GFR from MDRD and the CKD-EPI formulae are expressed as GFR in mL/min per 1.73 m^2^. Age is expressed in years, body weight in kg, and plasma creatinine in *μ*mol/L.

#### 2.7.1. Cockcroft-Gault Formula

(140 − age) × body weight/plasma creatinine × 72  (multiply by 0.85 if female) [26].

For comparison with the prediction of other formulas, the predicted creatinine clearance by Cockcroft-Gault was normalized per 1.73 m^2^ of BSA using the formula of Du Bois [23] to allow easier comparison between individuals.

(4) $$\begin{array}{c} =\frac{\left(140-\text{age}\right)\times \left(\text{body weight }\left(\text{multiply by }0.85\text{ if female}\right)/\text{plasma creatinine}\times 72\right)\times 1.73}{\text{BSA}}. \end{array}$$

### 2.8. Statistical Analysis

Statistical analysis was carried out using the Statistical Package for the Social Sciences (SPSS), version 21.0. Categorical data were presented as frequencies and percentages (in parenthesis). Continuous variables were expressed as the mean plus/minus standard deviation of the mean (mean ± SD) and as a 95% confidence interval. A Krouwer plot [27] was utilized to analyze the agreement between two sets of formulae. A *p* value < 0.05 was considered statistically significant.

## 3. Results

**Figure 1:**
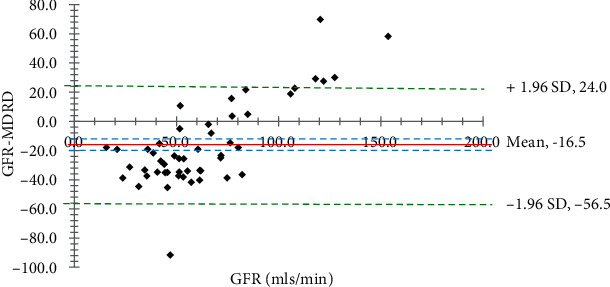
A Krouwer plot to compare measured GFR and MDRD predictive formula. The solid red horizontal line shows the mean difference, dotted blue lines show the 95% confidence interval of the mean difference (4.10), green dotted lines show limits of agreement (±1.96 standard deviation of the differences), and the GFR axis shows the line of equality (zero difference). The bias between the GFR and MDRD methods is statistically significant (*p* < 0.05).

**Figure 2:**
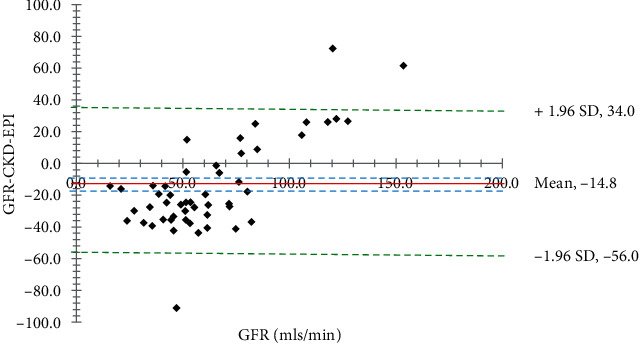
A Krouwer plot to compare the measured GFR with the CKD-EPI method. The solid red horizontal line shows the mean difference, dotted blue lines show the 95% confidence interval of the mean difference (4.10), green dotted lines show limits of agreement (±1.96 standard deviation of the differences), and the GFR axis shows the line of equality (zero difference). The bias between the GFR and CKD-EPI methods is statistically significant (*p* < 0.05).

**Figure 3:**
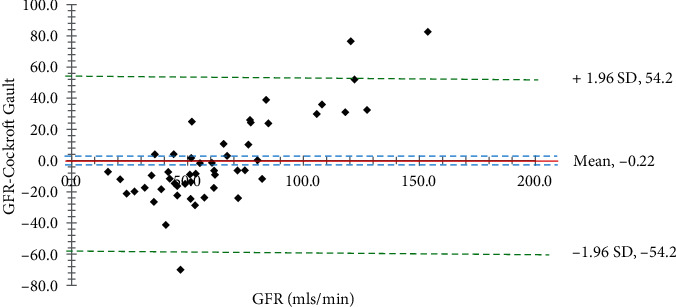
A Krouwer plot to compare the measured GFR with the Cockcroft-Gault method. The solid red horizontal line shows the mean difference which is almost on the GFR-axis, dotted blue lines show the 95% confidence interval of the mean difference (3.92), green dotted lines show limits of agreement (±1.96 standard deviation of the differences), and GFR-axis shows the line of equality (zero difference). The bias between the GFR and Cockroft methods is not statistically significant (*p* > 0.05).

Fifty (50) community dwellers (18 men and 32 females) with T2DM were recruited. The age of participants ranged from 30 to 70 years old, and their average age (±SD) was 57.4 ± 10.6 years. The vast majority of people tested did not have any detectable levels of glucose (74%) or protein (92%) in their urine (Table 1). Participant's mean BMI, FPG, serum creatinine, and urine creatinine were, respectively, 27.8 ± 4.1 kg/m^2^, 9.0 ± 3.1 mmol/L, 95.6 ± 29.1 *μ*mol/L, and 6.1 ± 2.2 mmol/L (Table 2). The estimated GFR for CKD-EPI, MDRD, and CG were 77.9 ± 21.3, 79.6 ± 20.2, and 63.3 ± 18.8, respectively. Krouwer plots to compare the predictive formulas with the measured GFR are shown in Figures 1–3. The bias between the measured GFR versus the CKD-EPI and MDRD methods was statistically significant (*p* < 0.05). Only the Cockroft-Gault showed nonsignificance (*p* > 0.05) with the measured GFR.

## 4. Discussion

Several formulae are used to estimate GFR in different clinical settings. There have been conflicting reports regarding the predictive formula that either provides the most accurate estimate of the GFR or corresponds exceptionally well with the GFR that has been measured [28–30]. In this observational study, our objective was to determine which predictive GFR formula was most comparable to the “traditional” creatinine clearance (GFR), which involves a 24-hour urine collection among a cross-section of patients with T2DM. This study focused on 3 GFR estimation formulae: MDRD, CKD-EPI, and CG. Even though these formulae have been validated in some settings, it is unclear which one provides the most accurate estimation of GFR. Our findings give insight into the degree to which these formulae are true when applied to a T2DM cohort in Ghana, a sub-Saharan African country.

In this study, we used the Krouwer plot instead of the Bland-Altman method to evaluate the agreement between two measurement methods for continuous outcomes. The Krouwer plot was selected for its perceived higher precision and to mitigate any potential bias from measurement errors. The average of all the differences of the estimates in this study, which is supplied by the *t*-test inferential statistics (sometimes referred to as the bias), and the 95% limits of agreement are the two primary pieces of information that are offered by a Krouwer plot (Figures 1–3). In addition, the capacity to display a link between the value being analyzed and the error introduced by the measurement is an intriguing quality of this particular plot [31, 32]. For instance, Michels et al. in a previous study showed, with the use of a Bland-Altman plot, that the MDRD formula overestimated the genuine measured GFR among higher GFR values, although this was not the case for lower GFR values [33].

The average measured GFR in this study was 64.8 ± 28.7, which was not significantly different from the estimated value of 63.3 ± 18.8 with the CG formula. A Krouwer plot to compare the measured GFR with the CG formula showed the smallest mean bias. This result implies that the CG formula may be the most appropriate alternative GFR estimate among Ghanaians with T2DM. The results of this study corroborate earlier studies [34, 35]; nevertheless, they disagree with the results of other studies [36, 37]. The CG was created in a cohort of mostly nonobese male individuals of various ages, weights within the 10% range of fat-free body mass, and normal kidney function. As a result, CG estimates are especially trustworthy at GFR values greater than 60 mL/min. Unlike the MDRD and CKD-EPI, body weight is included as a variable because it represents a rough approximation of muscle mass [34]. In a large diabetic cohort of 24,516 adults, CG was overestimated, while CKD-EPI was understated with the least bias and maximum accuracy for the MDRD. These formulas appear to be population-driven, with each having its own set of characteristics, advantages, and disadvantages in terms of application [36]. These formulas appear population-driven, with each formula having peculiarities and exhibiting merits and demerits in its usage.

The CG formula substitutes creatinine clearance for GFR in its estimations. The GFR and renal tubular secretion are both important components to achieving clearance of the endogenous substrate (creatinine) from the serum. Both the diet and the amount of muscle mass are critical components in producing endogenous creatinine. Muscle mass can be affected by several factors, including age, gender, total body weight, and the presence or absence of comorbidities [38, 39]. In the process of making creatinine in the body, diet plays a significant role as well [40].

Concerns have been raised over the use of a patient's total body weight in the assessment of CG, especially in the case of obese individuals. This is because research has shown that an increase in muscle mass is more strongly associated with a rise in lean body weight as opposed to an overall rise in total body weight [41, 42]. The fact that the patients' average body mass index was 27.8 ± 4.1, on the other hand, suggests that the greater majority of the study population did not suffer from obesity. This probably is one of the contributing factors that helped explain why there was less bias. In addition, some studies have shown that CG is an accurate predictor of GFR in individuals older than 12 years who are neither overweight nor obese [43, 44]. However, when the observed GFR values are low, the CG formula tends to yield findings that are optimistic [45]. Due to the limitations of our investigation, we were unable to verify that this is the situation.

In light of the difficulties associated with CG calculations, the MDRD was formulated as a four- and six-variable function that takes into consideration age, gender, serum creatinine, blood urea nitrogen, albumin, and race [46, 47]. To construct the MDRD formula, a sizeable patient sample as well as radiolabelled iothalamate, which is a more accurate exogenous substrate for measuring GFR, are used [48]. It has been stated that the MDRD is the most trustworthy clinical estimate of GFR in individuals who have a GFR of less than 60 mL/min/1.73 m^2^ [49]. The average measured GFR that we found in our study was 64.8 ± 28.7, and thus, we were not surprised to see the bias. Previous studies have reported that the MDRD formula produces an overestimate at higher GFR values [50, 51]. However, in the current investigation, we did not find any evidence of this.

The CKD-EPI formula, which was also established very recently, permits better determination of the GFR throughout the full range, not just for values below 60 mL/min/1.73 m^2^, as is the case with other formulae [52, 53]. Despite this, the group that was utilized to construct this new formula was predominantly individuals in their twenties or thirties who had a GFR of 70 mL/min/1.73 m^2^ [52]. There is a paucity of data on using the CKD-EPI formula for elderly adults. Since the average age of our study population was 57.4 ± 10.7, it is not far-fetched that the bias between the measured GFR and CKD-EPI methods was statistically significant. We could not establish an association that was satisfactory between the MDRD and CKD-EPI formulae and the GFR that was assessed in the “conventional” manner. Despite this, the projected values for MDRD and CKD-EPI were quite similar (79.6 ± 20.2 and 77.9 ± 21.3, respectively). In diabetic patients, the results of previous research have demonstrated that the MDRD and CKD-EPI formulae function similarly to one another in terms of accuracy [36, 54]. In a large data set of 3896 participants, the CKD-EPI was more accurate than the MDRD in determining GFR [55].

### 4.1. Limitations

There are several limitations to this study. We did not study other comorbidities of the research population, as these comorbidities could alter eGFR. Additionally, the population size that was used in our investigation was relatively small when compared to other studies that had a significantly larger number of participants. Thus, we were limited in performing within-group evaluations. This study, however, could serve as a basis for scaling up such research in a larger population. Nonetheless, in a resource-poor setting where access to quality healthcare remains a challenge, our findings could impact the management of renal conditions in T2DM. They could serve as a basis for further research in Ghana and beyond.

## 5. Conclusion

The primary finding of this study was that the CG predictive formula provided an accurate alternative GFR estimate comparable to that obtained using the traditionally measured GFR among a cohort of patients in Ghana with T2DM.

### 5.1. What Is Already Known on the Topic

- Glomerular filtration rate (GFR) is an important index in determining and monitoring a decline in renal clearance
- Several mathematical models have been introduced as alternatives to the traditionally measured GFR
- There have been conflicting reports about the predictive model that delivers the best accurate estimate of the measured GFR

### 5.2. What This Study Adds

- The Cockroft-Gault predictive formula provided the most accurate alternative GFR estimate among a cohort of type 2 diabetic patients in Ghana

## Figures and Tables

**Table 1:** Sociodemographic and clinical parameters of the study population.

Parameter	Frequency (*n* = 50) (%)
Gender	
Males	16 (32.0)
Females	34 (68.0)
Urine protein	
Yes	4 (8.0)
No	46 (92.0)
Urine glucose	
Yes	13 (26.0)
No	37 (74.0)
Positive urine glucose	
+1	2 (15.4)
+2	6 (46.1)
+3	4 (30.8)
+4	1 (7.7)

Clinical data (categorical variables) of participants are shown in Table 1. % is the percentage.

**Table 2:** Clinical and biochemical parameters of the study population.

Parameter	All (*N* = 50)	Males (*n* = 16)	Females (*n* = 34)	*p* value
Age (yrs)	57.4 ± 10.7	59.7 ± 13.3	56.4 ± 9.3	0.3271
BMI (kg/m^2^)	27.8 ± 4.1	27.4 ± 3.7	27.9 ± 4.3	0.6772
Urine creat (mmol/L)	6.1 ± 2.2	3.7 ± 1.4	6.0 ± 2.1	0.0003
Mean urine vol. (L)	1.7 ± 4.2	1.4 ± 0.5	1.4 ± 0.4	0.5353
Serum creat. (*μ*mol/L)	95.6 ± 29.1	115.0 ± 39.5	86.5 ± 16.8	0.0008
FPG (mmol/L)	9.0 ± 3.1	8.7 ± 2.0	9.2 ± 2.7	0.4985
Measured GFR	64.8 ± 28.7	58.1 ± 29.6	65.5 ± 28.5	0.3979
CKD-EPI	77.9 ± 21.3	77.9 ± 27.9	78.0 ± 17.9	0.9871
MDRD	79.6 ± 20.2	81.6 ± 26.7	78.6 ± 16.7	0.6290
Cockroft-Gault	63.3 ± 18.8	59.1 ± 21.8	65.3 ± 16.1	0.2756

In Table 2, the clinical and biochemical features of study participants are shown. The data are provided as means and standard deviations, with a significance threshold of 0.05. BMI: body mass index; FPG: fasting plasma glucose; CKD-EPI: Chronic Kidney Disease Epidemiology; MDRD: Modification of Diet in Renal Disease.

## Data Availability

The corresponding author will make all data sets available to anyone upon reasonable request
